# Affective Profiles and Anxiety or Non-Anxiety-Related Reasons for School Refusal Behavior: Latent Profile Analysis in Spanish Adolescents

**DOI:** 10.3389/fpsyg.2021.666218

**Published:** 2021-03-25

**Authors:** Carolina Gonzálvez, Ángela Díaz-Herrero, María Vicent, Ricardo Sanmartín, Aitana Fernández-Sogorb, Cecilia Ruiz-Esteban

**Affiliations:** ^1^Department of Development Psychology and Teaching, University of Alicante, Alicante, Spain; ^2^Department of Development Psychology and Education, University of Murcia, Murcia, Spain

**Keywords:** affective profiles, positive affect, negative affect, school refusal behavior, anxiety, latent profile analysis, adolescence

## Abstract

Little has been studied on the relationship between affect and school problems related with attendance. This study aims to identify different affective profiles and to determine whether these profiles differ from each other based on the four functional conditions of school refusal behavior. Participants comprised 1,816 Spanish adolescents aged 15–18 years (*M* = 16.39; *SD* = 1.05). The Positive and Negative Affect Schedule for Children-Short Form and the School Refusal Assessment Scale-Revised for Children (SRAS-R-C) were administered. Latent profile analysis revealed five affective profiles: low affective profile, self-fulfilling profile, low positive affect profile, self-destructive profile, and high affective profile. The self-destructive profile revealed the highest average scores in the first three factors of the SRAS-R-C, whereas the high affective profile reached the highest average score in the fourth factor. On the contrary, the self-fulfilling profile obtained the lowest average scores in the first two factors of the SRAS-R-C, whereas the low affective profile revealed the lowest average scores in the last two factors. Findings suggest the relevance of developing more adaptative affective profiles, such as the self-fulfilling profile, which would contribute to diminishing school attendance problems.

## Introduction

Affect, understood as the central core of emotions, plays an essential role in the human experience (Díaz-García et al., 2020). Its study is a complex subject as, among other aspects, physiological mechanisms, cognitive components, behavioral expressions, and social and cultural conditioners are all involved (Alcalá et al., 2006; Buzzai et al., 2020).

Scientific evidence supports a two-dimensional model in the basic structure of affect, distinguishing two large independent dimensions called positive affect and negative affect (Watson and Tellegen, 1985; Watson et al., 1988; Watson and Clark, 1994; Padrós et al., 2012). High positive affect is characterized by energy, joy, concentration, interest, enthusiasm, and rewarding participation, while low positive affect alludes to apathy, slowness, and lethargy (Watson and Clark, 1984). In contrast, high negative affect represents a general discomfort dimension that includes a variety of moods such as anger, guilt, fear, dislike, and nervousness. Calm and serenity would be components of low negative affect (Watson et al., 1988; Clark et al., 1994). These two dimensions of the affective structure can be conceptualized either as affective states or as somewhat stable temperamental–emotional dispositions (Watson and Clark, 1994; Sandín et al., 1999).

Affective functioning, based on the tripartite model of emotion, has been associated with the clinical symptoms and disorders of anxiety and depression (Clark and Watson, 1991). In this regard, it is widely indicated that anxiety and depression share high levels of negative affect, while depression is only characterized by low levels of positive affect (Watson and Tellegen, 1985; Clark et al., 1994). However, more recent studies challenge these claims by finding, in studies with non-clinical samples, that both anxiety and depression are characterized by high negative affect and low positive affect (e.g., Domaradzka and Fajkowska, 2019). They also find that social anxiety correlates positively with negative affect and negatively with positive affect (Anderson et al., 2010). Furthermore, some researchers have highlighted that negative affect is related not only to internalizing disorders but also to externalizing disorders (Loney et al., 2006; Baldwin and Dadds, 2008). In these studies, positive correlations were found between negative affect and behavior problems and attention deficit hyperactivity disorder in children between 8 and 18 years old.

To evaluate affect, Watson et al. (1988) designed the Positive and Negative Affect Schedule (PANAS). This is a tool widely used by the scientific community and has been shown to possess adequate psychometric properties in Spanish adolescents (Sandin, 2003; Ebesutani et al., 2012; Ortuño-Sierra et al., 2015, 2019; Sanmartín et al., 2020). This instrument conceptualizes positive and negative affect as independent orthogonal dimensions that can be categorized at a high or low level. Based on this scale, Norlander et al. (2002, 2005) developed the affective profiles model by classifying people into four profiles, which they named self-fulfilling (high positive affect and low negative affect), high affective (high positive affect and high negative affect), low affective (low positive affect and low negative affect), and self-destructive (low positive affect and high negative affect). These profiles were based on the division of the median affect scores. These profiles have been subsequently replicated in studies carried out with samples of different ages and nationalities. An example of this is the works of Sanmartín et al. (2018a,b, 2020), who, through cluster analysis, identified these same profiles in Spanish children and Ecuadorian adolescents.

This typology of affective profiles has been used in subsequent research examining, above all, their relationships with variables related to psychological adjustment. As such, studies conducted in adolescents have focused on analyzing the differences between affective profiles with respect to various measures of psychological well-being, personality, social anxiety, and self-regulation. In this regard, Garcia and his collaborators (Garcia and Siddiqui, 2009; Garcia et al., 2010, 2012; Garcia, 2012; Garcia and Archer, 2012; Schütz et al., 2014) found in their work with Swedish adolescents that those with a self-fulfilling profile, compared to the other profiles, reported higher life satisfaction, greater psychological well-being, fewer depressive symptoms, less stress, and higher scores for personality traits related to personal characteristics such as autonomy, responsibility, self-acceptance, internal locus of control, and self-control. Similarly, they observed that adolescents with a low affective profile, compared to those with a self-destructive profile, reported being more satisfied with life and experiencing higher levels of psychological well-being (Garcia and Siddiqui, 2009). In addition, the results of Garcia's (2012) study indicated that adolescents with high affective and self-destructive profiles, compared to those with low affective and self-fulfilling profiles, presented higher scores in neuroticism. This finding may be due to the fact that high affective and self-destructive profiles have high levels of negative affect as a common characteristic (Watson and Clark, 1984).

Similar results were corroborated in Iranian (Garcia and Moradi, 2013) and Italian (Di Fabio and Bucci, 2015) adolescents, where it was again observed that those categorized as having the self-fulfilling profile showed higher levels of life satisfaction, psychological well-being, self-esteem, and optimism. More recently, in a study carried out with Ecuadorian adolescents, Sanmartín et al. (2020) found that students categorized as having the self-fulfilling profile showed the lowest scores for social anxiety, while those categorized as having the self-destructive profile obtained the highest scores. In summary, most research highlights that the self-fulfilling profile is related to a greater psychological adjustment, while the self-destructive profile is associated with more maladaptive variables.

Feelings of negative affectivity are also present in school refusal behavior, understood as a child's refusal to attend school and/or their persistent difficulty with staying in class throughout the school day (Hendron and Kearney, 2011). A widely used classification system to analyze this behavior is the functional model of assessment proposed by Kearney and Silverman (1990). These authors begin their work with the fact that school refusal behavior can be motivated by four factors or functional conditions: (1) avoidance of negative affectivity caused by stimuli related to the school environment; (2) escape from aversive social and/or evaluative situations such as exams; (3) seeking of attention from significant others; and (4) seeking of tangible reinforcements outside the school environment, such as dedicating the school day to activities that turn out to be more appealing, like being with friends or playing video games. Based on this model, Kearney and Silverman (1993) designed the School Refusal Assessment Scale (SRAS) and its revised version, the School Refusal Assessment Scale-Revised for Children (SRAS-R-C; Kearney, 2002), which allow for the measurement of the relative strength for these four functional conditions in each particular case. From this, specific prevention and treatment strategies are able to be established. As Haight et al. (2011) indicated, prescriptive treatments include child-based psychoeducation, somatic control exercises, cognitive restructuring, and exposure-based practice for the school refusal behavior based on the first two factors of the SRAS-R-C, as well as parent-based contingency management (factor 3) and family-based contingency contracting and communication skills training (factor 4).

School refusal behavior has been associated with both internalizing and externalizing behavior problems, as found in numerous studies (e.g., Egger et al., 2003; Kearney and Albano, 2004; Nayak et al., 2018). In summary, positive and statistically significant relationships with anxiety disorders and depression have been found in adolescents whose school refusal behavior is based on the first three factors of the functional model (Kearney and Albano, 2004; Haight et al., 2011; Richards and Hadwin, 2011; Gonzálvez et al., 2020). In contrast, significant relationships with externalizing behavior disorders have been observed in adolescents whose school refusal behavior is motivated by obtaining tangible reinforcements outside of school (Kearney and Albano, 2004; Haight et al., 2011). These findings highlight the relevance, above all, of negative affectivity in the first three factors in the functional model for school refusal behavior. This fact would also be supported by the established associations between negative affect and anxiety as well as depression disorders, according to the tripartite model of emotion.

Despite this, there are scarcely any studies in the scientific literature that have analyzed the relationships between school refusal behavior and affective functioning. Furthermore, with the exception of an investigation carried out by Sanmartín et al. (2018a) in Spanish children, the existing studies have focused only on analyzing the relationships between the dimensions of positive and negative affect and the factors of the functional model, without considering the affective profiles model. In this regard, in the study by Higa et al. (2002), which sought to evaluate the psychometric properties of SRAS, positive and significant correlations were found between negative affect (measured through the Affect and Arousal Scale for Children, AFARS, Chorpita et al., 2000) and the first three SRAS factors, not being significant for the fourth factor. This study was conducted on 30 American children ranging in age from 8.11 to 17.8 years. Similarly, in another paper that also aimed to analyze the psychometric properties of SRAS-R-C, Gonzálvez et al. (2016) found in a sample of 1,078 Spanish children between 8 and 11 years of age that negative affect (evaluated by PANAS) correlated positively and significantly with the first three SRAS-R factors. In contrast, positive affect showed negative and significant correlations with the first two factors and positive and significant correlations with the fourth SRAS-R-C factor.

These same relationship patterns between the affect dimensions of the 10-item PANAS for Children (PANAS-C; Ebesutani et al., 2012) and school refusal behavior have also been found in other studies. Inglés et al. (2016) found, in a sample of 476 Spanish children between 8 and 12 years, that negative affect showed positive and significant correlations with the first three SRAS-R-C factors and did not find significant relationships with the fourth. The results of the study by Gonzálvez et al. (2018a) also indicate the same. In fact, in their study, the logistic regression analyses revealed that positive affect predicted negative and significantly high scores in school refusal for the first two factors, while it predicted positive and significantly high scores for SRAS-R factors 3 and 4. This study was conducted with a sample of 1,078 Spanish children between 8 and 11 years of age.

Finally, as already outlined, the only study carried out on this matter and based on the affective profiles model was that by Sanmartín et al. (2018a). Using a cluster analysis, these authors first attempted to verify the existence of the four affective profiles indicated by Norlander et al. (2002, 2005). Secondly, they sought to analyze the relationships of these profiles with school refusal behavior. They gave PANAS-C and SRAS-R-C to a sample of 1,575 Spanish students between the ages of 8 and 11. The cluster analyses corroborated the four affective profiles: self-fulfilling profile, high affective profile, low affective profile, and self-destructive profile. In addition, *post-hoc* comparisons highlighted that children with a self-destructive profile scored significantly higher on the first three SRAS-R-C factors compared to children with the other profiles. In contrast, the self-fulfilling profile showed significantly higher scores on the fourth SRAS-R-C factor compared to the low affective and self-destructive profiles.

In summary, the literature review shows that there are scarcely any studies that use rigorous methods to establish affective profiles. Most research conducted to date, except for the studies by Sanmartín et al. (2018a,b, 2020), has been based on the median-split technique to determine the four affective profiles. This fact has been criticized by authors such as Garcia et al. (2015), who have questioned its arbitrary nature and propose cluster analysis as a more appropriate statistical methodology. On the other hand, it is observed that there is only one study carried out with Spanish children that, after establishing affective profiles by means of cluster analysis, analyzes their relationships with school refusal behavior. There are no studies in the adolescent population. Therefore, it would be important to analyze the relationships between affective profiles and school refusal behavior in adolescents in order to detect possible protective and/or risk factors in this age group. This would subsequently allow for the development of prevention and intervention strategies to reduce the incidence of this problem in the school environment.

The two objectives of this research are proposed with consideration to these limitations and proposals. The first objective is to verify the existence, by means of latent profile analysis (LPA), of the four affective profiles using a combination of the positive and negative affect dimensions evaluated through PANAS. LPA, unlike cluster analysis, is a method that fits a statistical model to the data and classifies each person in the most likely group based on their responses to a set of observed variables. It is a tool that focuses on the similarities and differences between individuals rather than the relationships between variables and is considered a more accurate technique than cluster analysis (Berlin et al., 2014). Based on Norlander et al. (2002, 2005), it is expected to identify four affective profiles: self-fulfilling profile, low affective profile, high affective profile, and self-destructive profile. Once the affective profiles have been identified, the second objective is to analyze whether there are statistically significant differences between the profiles with respect to the four motivating factors of school refusal behavior in SRAS-R. Higher scores on the first three SRAS-R-C factors were expected in students belonging to the self-destructive profile (Sanmartín et al., 2018a).

## Methods

### Participants

The study sample consisted of 1,816 Spanish adolescents (51.3% boys) whose ages ranged from 15 to 18 years (*M* = 16.39, *SD* = 1.05). Table 1 shows the sample's distribution by gender and age. All participants were typically developing adolescents with no psychological, behavioral, or linguistic problems. The initial sample included 1,899 students from Alicante and Murcia. However, 83 students were excluded either because they did not give the written informed consent from their parents (*n* = 49) or because there were errors or omissions in the completed questionnaires (*n* = 34). The final sample comprised a normative sample of 1,816 students. The chi-square test of homogeneity in the frequency distribution revealed the absence of statistically significant differences between the sex and age groups (χ^2^ = 3.74; *p* = 0.29). Socio-economic distribution corresponded mainly to the average level (21% medium-low, 66% medium, and 13% medium-high) according to the parents' or legal guardians' academic level.

**Table 1:** Distribution of the sample by sex and age.

**Sex**	**Age**				**Total**
	**15**	**16**	**17**	**18**	
Boys	217	284	246	184	931
	11.9%	15.6%	13.5%	10.1%	51.3%
Girls	235	261	237	152	885
	12.9%	14.4%	13.1%	8.4%	48.7%
Total	452	545	483	336	1816
	24.9%	30%	26.6%	18.5%	100%

### Measures

The PANAS-C-Short Form (PANAS-C-SF; Ebesutani et al., 2012) is a self-report measure for children and adolescents between 6 and 18 years that assesses positive and negative affect. It is a 10-item questionnaire made up of two subscales measuring the positive (joyful, lively, happy, energetic, and proud) and the negative (depressed, angry, fearful/scared, afraid, and sad) dimensions of affectivity present during the preceding weeks of it being completed. The 10 items are rated on a 5-point Likert scale (ranging from 1 = very slightly or never to 5 = very much). The Spanish version of this report developed by Sanmartín et al. (2018b), which remains unchanged from the original version, was used in this study. The two subscales showed appropriate internal consistency values in the original study (positive affect.86; negative affect.82) and also in this study (positive affect.82; negative affect.71).

The SRAS-R-C (Kearney, 2002) is a self-report measure for children and adolescents between 8 and 18 years. The SRAS-R-C assesses the relative influence of four functional conditions of school refusal behavior: (1) avoidance of stimuli that provoke negative affectivity [e.g., “How often do you have bad feelings about going to school because you are afraid of something related to school (for example, tests, school bus, teacher, fire alarm)?”]; (2) escape from aversive social and/or evaluative situations (e.g., “How often do you stay away from school because it is hard to speak with the other kids at school?”); (3) pursuit of attention from significant others (e.g., “How often do you feel you would rather be with your parents than go to school?”); and (4) pursuit of tangible reinforcement outside of school [e.g., “When you are not in school during the week (Monday to Friday), how often do you leave the house and do something fun?”]. Through a 7-point Likert scale (from 0 = never to 6 = always), the scale includes 24 items (six items for each of the four dimensions). In this study, we used the Spanish version of the report developed by Gonzálvez et al. (2016), which is made up of 18 items from the 24 originally proposed. The four subscales showed appropriate internal consistency values in the original study that ranged between 0.78 (factor 3) and 0.59 (factor 4) (Kearney, 2002). The Spanish version reported values between 0.87 (factor 3) and 0.70 (factor 1) (Gonzálvez et al., 2016). In this study, the coefficients of internal consistency were 0.64, 0.73, 0.78, and 0.56 for factors 1, 2, 3, and 4, respectively, using the Spanish version of SRAS-R-C (Gonzálvez et al., 2016).

### Procedure

First, an interview was conducted with the principals from the 19 high schools with the purpose of explaining the aims of the study and to ask for their collaboration. Most principals were in favor of participating, and finally, 16 public and private high schools located in Alicante and Murcia cooperated. Once the participants' voluntary collaboration was given, they completed the two questionnaires. The measures were completed voluntarily in the high schools' classrooms in a 30-min session. The order of application of PANAS-C-SF and SRAS-R-C was as follows: half of the subjects in each group first filled the measure on affect and then the scale on school refusal behavior, while the other half filled out the questionnaires in the reverse order. The Ethics Committee of the University of Alicante (code of ethics: UA-2017-09-05) approved the study, and the standards established by the Declaration of Helsinki (Rickham, 1964) were followed.

### Statistical Analyses

Firstly, correlations between the positive and negative affect and the four conditions of school refusal behavior were tested using Pearson's product–moment correlation coefficient. Values equal to or >0.10 and <0.30 indicated a small or weak correlation. Values >.30 indicated a moderate correlation, while values >0.50 indicated a high correlation (Cohen, 1988). For this, the SPSS 24 program was used.

Secondly, an LPA was performed to identify the cluster solutions for the two-factor conceptualization of affectivity. To determine the most adequate class solution, a series of LPA models were applied. The classification accuracy of each solution was examined using seven fit statistics criteria to evaluate the models: the Akaike information criterion (AIC), the Bayesian information criteria (BIC), the BIC adjusted, the Vuong–Lo–Mendell–Rubin likelihood ratio test (LRT), the LRT adjusted, the bootstrap likelihood ratio test (BLRT), and entropy. The model with the lowest BIC and AIC values was preferred. Regarding LRT and BLRT statistics, a *p*-value below 0.05 indicated that the estimated *k*-class model was better than the (*k* – 1)-class model, which was therefore rejected in favor of a model with at least *k* classes (Wang and Wang, 2012). In addition, entropy was used as a criterion for the quality of class membership classification, where a score closer to 1 was preferred. Finally, the index of size was considered, including the best model with at least 1% of the sample (Tein et al., 2013). Beyond these indices, theoretical feasibility and psychological significance, together with the maximization of the inter-class differences of each of the groups, should be considered in selecting the best model. Mplus version 8 was used in this study because it provides these statistics (Muthén and Muthén, 2012).

Finally, to test group differences, a multivariate analysis of variance (MANOVA) was used to compare the differences in the school refusal behavior dimensions between the affective profiles identified. The partial eta-squared index ($\eta_{\text{p}}^{2}$) and *post-hoc* tests (Bonferroni's method) were performed to identify which groups had statistically significant differences between them. Likewise, the effect size was calculated using the *d* index to obtain the magnitude of the differences observed (Cohen, 1988). The *d* index was interpreted as follows: values between 0.20 and 0.49 indicated a low effect size; values between 0.50 and 0.79, a moderate effect size; and values above 0.80, a high effect size. SPSS version 24 was used in this study to analyze these data.

## Results

### Affect and School Refusal Behavior's Correlations

Correlations between the positive and negative affect and the four conditions of school refusal behavior were largely statistically significant and weak in all cases (see Table 2). The four school refusal behavior dimensions positively correlated with the Negative Affect although the fourth factor of the SRAS-R-C does not have a significant effect size. On the other hand, the negative reinforcement conditions, which are the first two factors of the SRAS-R-C, negatively correlated with the Positive Affect, whereas the tangible rewards dimension, which is the fourth factor of the SRAS-R-C, positively correlated with the Positive Affect.

**Table 2:** Correlations between affect and school refusal behavior.

	**Positive affect**	**Negative affect**
SRAS-R-C Factor 1	−0.14^**^	0.33^**^
SRAS-R-C Factor 2	−0.11^**^	0.26^**^
SRAS-R-C Factor 3	0.01	0.19^**^
SRAS-R-C Factor 4	0.13^**^	0.08^*^

* *p < 0.01*;

** *p < 0.001; SRAS-R-C, School Refusal Assessment Scale-Revised for Children*.

### School Refusal Behavior Profiles

Latent profile models containing between two and seven classes were fit to the data. Table 3 shows the model fit indices for each LPA. The LRT and BLRT indicated that the two-class solution and the five-class solution fit better than the other models. However, the five-class solution was deemed superior to the two-class solution due to its lower AIC and BIC values. Although the six- and seven-class solutions revealed slightly lower AIC and BIC values, the five-class solution revealed better entropy scores and more significant values for BIC and LRT indices. When all the criteria were combined, the fifth model was selected as the best fitting.

**Table 3:** Data fit of all models.

**Models**	**AIC**	**BIC**	**BIC adjusted**	**LRT**	**LRT adjusted**	**BLRT**	**Entropy**	**Size**
2	9,996.744	10,035.158	10,012.919	<0.001	<0.001	<0.001	0.673	0
3	9,973.019	10,027.896	9,996.127	0.2204	0.2317	<0.001	0.590	0
4	9,951.120	10,022.461	9,981.161	0.0888	0.0938	<0.001	0.662	0
5	9,898.633	9,986.437	9,935.606	<0.001	<0.001	<0.001	0.702	0
6	9,894.605	9,998.872	9,938.510	0.0030	0.0037	<0.001	0.719	1
7	9,867.086	9,987.816	9,917.923	0.0032	0.0038	<0.001	0.714	1

LRT, Vuong–Lo–Mendell–Rubin likelihood ratio test; BLRT, bootstrap likelihood ratio test.

**Figure 1:**
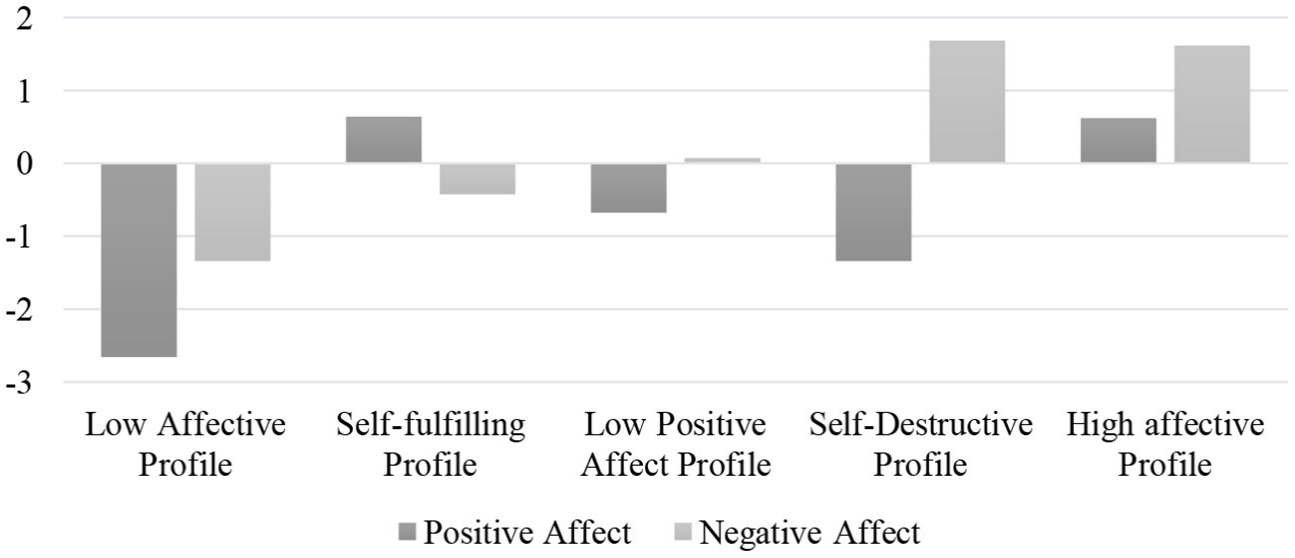
Affective profiles.

Figure 1 illustrates the five-class solution model. Class 1 consisted of 2.2% of the sample (*n* = 40) and represents individuals with low scores in positive and negative affect. This profile was referred to as the “low affective profile.” Class 2 consisted of 49.5% of the sample (*n* = 899) and represents individuals with high scores in positive affect and relatively low scores in negative affect. This profile was labeled as the “self-fulfilling profile.” Class 3 consisted of 38.4% of the sample (*n* = 698) and was labeled the “low positive affect” because it consisted of individuals with low levels of positive affect. Class 4 consisted of 4.7% of the sample (*n* = 86) and represents adolescents with low levels of positive affect and high levels of negative affect. This model was labeled the “self-destructive profile.” Finally, class 5 was referred to as the “high affective profile” due to its high scores in positive and negative affect and represents 5.1% of the sample (*n* = 93).

### Differences Between Affective Profiles and School Refusal Behavior

MANOVA was used to examine differences among the five affective profiles on the four functional conditions of school refusal behavior. Statistically significant differences were found among the latent profiles in the four functional conditions of school refusal behavior [Wilks' lambda = 0.878, *F*_(16, 1811)_ = 15.04; *p* < 0.001, η${}_{\text{p}}^{2}$ = 0.03]. The self-destructive profile showed the highest average scores in the first three factors of SRAS-R-C, whereas the high affective profile reached the highest average score in the fourth SRAS-R-C factor. On the contrary, the self-fulfilling profile obtained the lowest average scores in the first two SRAS-R-C factors, whereas the low affective profile revealed the lowest average scores in the last two factors of SRAS-R-C (see Table 4).

**Table 4:** Means and standard deviations obtained by the five clusters in SRAS-R-C dimensions.

**Dimensions SRAS-R-C**	**Low affectivity profile**		**Self-fulfilling profile**		**Low positive affect profile**		**Self-destructive profile**		**High affectivity profile**		**Statistical significance**	
	***M***	***SD***	***M***	***SD***	***M***	***SD***	***M***	***SD***	***M***	***SD***	***F_**(4, 1811)**_***	***$\eta_{p}^{2}$***
F1	14.40	9.20	13.37	5.69	15.35	5.81	20.52	8.73	17.79	7.68	37.82^*^	0.077
F2	13.41	7.23	12.33	5.01	13.78	5.61	17.00	7.47	16.78	7.83	26.17^*^	0.055
F3	12.40	7.34	16.29	6.93	17.02	6.84	20.09	9.16	18.95	8.12	11.92^*^	0.026
F4	14.89	9.01	21.09	7.51	20.18	6.64	21.37	7.64	23.06	7.85	10.56^*^	0.023

*SRAS-R-C, School Refusal Assessment Scale-Revised for Children; F1: Avoidance of stimuli that provoke negative affectivity; F2: Escape from aversive social and/or evaluative situations; F3: Pursuit of attention from significant others; F4: Pursuit of tangible reinforcement outside of school*.

* *p < 0.001*.

Table 5 presents the *post-hoc* comparisons with effect size values ranging from 0.23 and 1.19. The largest effect sizes have been found by comparing the self-fulfilling, self-destructive, and high affective profiles with the low affective profile, scoring the first three highest in the fourth SRAS-R-C factor with a large size effect, as well as the self-destructive and high affective profiles in the third SRAS-R-C factor. On the other hand, the self-destructive and the high affective profiles scored higher than the self-fulfilling profile on the first two SRAS-R-C factors with large and moderate effect sizes. Finally, differences with a large effect size have been found between the low positive profile and the self-destructive profile in which the latter scored higher in the first SRAS-R-C factor.

**Table 5:** Cohen’s *d* value for *post-hoc* contrasts between cluster groups on SRAS-R-C dimensions.

**Dimensions SRAS-R-C**	**Profiles 1–2**	**Profiles 1–3**	**Profiles 1–4**	**Profiles 1–5**	**Profiles 2–3**	**Profiles 2–4**	**Profiles 2–5**	**Profiles 3–4**	**Profiles 3–5**	**Profiles 4–5**
F1	-	-	−0.69	-	−0.34	−1.19	−0.75	−0.83	−0.40	0.33
F2	-	-	−0.49	−0.44	−0.23	−0.89	−0.83	−0.55	−0.51	-
F3	−0.56	−0.67	−0.89	−0.83	-	−0.53	−0.38	−0.43	-	-
F4	−0.82	−0.78	−0.80	−0.99	-	-	-	-	−0.42	-

SRAS-R-C, School Refusal Assessment Scale-Revised for Children; F1: Avoidance of stimuli that provoke negative affectivity; F2: Escape from aversive social and/or evaluative situations; F3: Pursuit of attention from significant others; F4: Pursuit of tangible reinforcement outside of school; Profile 1 = low affectivity profile; Profile 2 = self-fulfilling profile; Profile 3 = low positive affect profile; Profile 4 = self-destructive profile; Profile 5 = high affectivity profile.

## Discussion

This research has the objective of identifying the four affective profiles suggested by Norlander et al. (2002, 2005) in a large community sample of Spanish adolescents. Moreover, it seeks to determine whether there are statistically significant differences between the affective profiles with respect to the four functional conditions or factors that motivate school refusal behavior according to SRAS-R-C. This is a pioneering study as it was carried out in a Spanish adolescent population and because it applies the LPA technique to identify the profiles. In addition, it provides empirical evidence for how the profiles relate to school refusal behavior.

With respect to the first objective, the LPA distinguished five affective profiles: self-fulfilling profile (high positive affect and relatively low negative affect), low affective profile (low positive affect and low negative affect), high affective profile (high positive affect and high negative affect), self-destructive profile (low positive affect and high negative affect), and low positive affective profile (low positive affect). Four of these profiles (self-fulfilling profile, low affective profile, high affective profile, and self-destructive profile) coincided, to a large extent, with those established by Norlander et al. (2002, 2005), confirming partially the first hypothesis, although in the self-fulfilling profile the negative affect scores were relatively low rather than being low. These results were similar to those obtained in the research by Sanmartín et al. (2018a,b, 2020) in which the profiles were established through a cluster analysis. In contrast, the low positive affective profile had not been detected in previous studies and was made up solely of low positive affect scores. According to the tripartite model, this would be an affective profile related to depression.

Regarding the second objective of the study, the results revealed statistically significant differences between the different affective profiles in terms of school refusal behavior. Generally speaking, it was found that adolescents with a self-destructive profile showed the highest scores for the first three SRAS-R-C factors, compared to the other profiles, thereby supporting the second hypothesis. In contrast, the highest scores for the fourth factor were obtained by adolescents with a high affective profile. On the contrary, the lowest scores for the first two factors were obtained by the adolescents with the self-fulfilling profile, and the lowest scores for the last two factors, by those with the low affective profile. These data were supported by the analysis of effect sizes. Indeed, when comparing the self-fulfilling profile with the self-destructive and high affective profiles, in the first two SRAS-R-C factors, the effect sizes were high or moderate. Similarly, when comparing the low affective profile with the self-destructive and high affective profiles, in factors 3 and 4 of SRAS-R-C, the effect sizes were high.

Likewise, it was observed that adolescents who were categorized in the self-fulfilling, self-destructive, and high affective profiles showed higher scores in the fourth factor when compared with those with the low affective profile. The size of the effect was large. Finally, adolescents from the low positive affective profile, when compared to those from the self-destructive profile, showed lower scores on the first SRAS-R-C factor, with a high effect size. The rest of the comparisons between groups did not provide important results for the study, and in all cases, the effect sizes were small or moderate.

Consequently, based on these data, we can assert that adolescents belonging to the self-destructive profile were those who exhibited higher scores in the first three SRAS-R-C factors. In addition, as suggested by their comparison with the low positive affective profile, low positive affect is of great importance in the first factor. The first three SRAS-R-C factors consider that school refusal behavior is motivated by anxiety or discomfort that may be caused by stimuli related to the school environment and social situations which involve either assessment or separation from loved ones. In fact, these factors have shown comorbidity with anxious or depressive symptoms and anxiety and/or depression disorders (Kearney and Albano, 2004; Haight et al., 2011; Gonzálvez et al., 2020). Therefore, these statements are consistent with the relationships identified in other studies between high negative affect and low positive affect and anxiety and/or depression (Anderson et al., 2010; Domaradzka and Fajkowska, 2019). Similar results were seen in the work of Sanmartín and collaborators, where the self-destructive profile scored significantly higher on the first three SRAS-R-C factors, compared to the other profiles (Sanmartín et al., 2018a), and had higher scores for social anxiety (Sanmartín et al., 2020). These findings would also be in line with the data provided from the studies by Higa et al. (2002), Gonzálvez et al. (2016), and Inglés et al. (2016), where significant correlations were found between negative affect and the first three SRAS-R-C factors.

Paradoxically, the high affective profile seemed to show a similar pattern of results. As in the self-destructive profile, although to a lesser extent, adolescents in this profile showed high scores on the first two SRAS-R-C factors. This finding may be due to the fact that the high affective and self-destructive profiles have high levels of negative affect as a common characteristic, and it could be that this dimension shows a greater weight in this profile. In addition, the correlations made in our study between positive affect and the first two SRAS-R-C factors were negative. However, these data require further research for analysis.

In contrast, the lowest scores on the first two SRAS-R-C factors were obtained by adolescents who were classified with the self-fulfilling profile. This result would be, to some extent, supported by the negative and significant correlations between positive affect and the first two SRAS-R-C factors found in the study by Gonzálvez et al. (2016). Likewise, this finding is indirectly supported by the research carried out with adolescents (Garcia et al., 2012; Sanmartín et al., 2020) in which it was observed that the adolescents with the self-fulfilling profile showed lower scores in depressive symptoms and social anxiety, psychological variables related to school refusal behavior in these first two SRAS-R-C factors.

As for factors 3 and 4 of SRAS-R-C, where school refusal behavior is maintained by positive reinforcement (not attending school allows the young person to have the attention of parents or allows them to devote school time to activities that are more enjoyable and attractive to them), the lowest scores were obtained by students with a low affective profile. These statements could be said to be in line with the study by Garcia and Siddiqui (2009) in which it was highlighted that adolescents with a low affective profile, compared to self-destructive ones, reported being more satisfied with their lives and experienced higher levels of psychological well-being. In contrast, the highest scores on the fourth SRAS-R-C factor (related to truancy) were obtained by adolescents with a high affective profile. This result differs from that obtained in the study by Sanmartín et al. (2018a) where the highest scores with this factor were obtained by the self-fulfilling profile, although in our study, the adolescents in the self-fulfilling profile also showed high scores in this fourth functional condition. However, our results may be supported by other research in which positive relationships have been found both between negative affect and behavioral problems (Loney et al., 2006; Baldwin and Dadds, 2008) and between positive affect and the fourth SRAS-R-C factor (Gonzálvez et al., 2016, 2018a). Therefore, it seems that presenting high levels of positive and negative affect could be a risk factor leading to the development of truancy-related behavioral problems, in which the anxiety component is not present.

In short, our data highlight that the self-destructive profile is the most maladaptive affective profile in terms of school refusal behavior. In fact, adolescents who are characterized by fear, anger, nervousness, lack of interest, guilt, shame, and high temperamental sensitivity to negative stimuli, among other aspects, are more likely to experience school attendance problems when faced with certain school situations that cause them discomfort, anxiety, and/or depression. Likewise, the high affective profile seems to be related to the problem of truancy. In this case, the adolescents, together with characteristics related to discomfort, would show enthusiasm, joy, energy, interest, and motivation, which, perhaps, would incite them to look for other activities that they find more fun or appealing outside the school environment during class time. Based on these findings, it appears that the influence of positive affect as a possible protective factor of school refusal behavior is complex and depends on the cause that justifies or motivates the behavior and may even, in some cases, be a reinforcer of such behavior (Gonzálvez et al., 2018a). In these adolescents, it would be important to work on the rational interpretation of their behavior and to reflect on the consequences linked to truancy.

Despite its contributions, this research has several limitations that should be highlighted. Firstly, the absence of studies that examine the configuration of affective profiles through LPA makes it difficult to contrast the empirical evidence found in this research. Secondly, the comparison of the results found in this study with those of other works is complex because there is no research with adolescents on this subject. In addition, from a preventive approach, adolescents who attend school regularly participated in this study, but it would be interesting to compare these findings with students who have school attendance problems. Thirdly, the findings cannot be generalized to other cultures or age groups different from the study's reference population. Given the scarcity of studies on this topic, it would be necessary to carry out further studies that analyze the relationships between these variables to verify whether these results coincide with those obtained in samples of other age ranges and other nationalities. Fourthly, the universal nature of the study does not allow us to make causal inferences. This could be solved by carrying out longitudinal studies and using structural equation models. Finally, another limitation of our study is that only self-report measures have been used. It would be advisable, for future works, to adopt a multi-method (e.g., interviews and self-registrations) and multi-source evaluation perspective (e.g., parents and teachers).

In conclusion and despite these restrictions, this study is of great relevance since it provides the first real results for the adolescent population regarding the relationships between affective profiles and school refusal behavior. The identification of associations between different affective profiles and school attendance problems could provide useful information for the design and development of prevention or treatment programs in cases of school refusal. Several studies point out the relevance of early identification of school attendance problems due to their short- and long-term consequences. In the short term, academic performance, attitudes toward school, and social achievement can be affected, whereas the long-term consequences may negatively influence the students' academic, psychological, and social development (Munkhaugen et al., 2017). Numerous studies noted that high rates of emotional problems, such as anxiety, depression, and stress, are common in students with school refusal behavior (Kearney and Albano, 2004; Gonzálvez et al., 2018b); also, associations with higher levels of cyberbullying (Delgado et al., 2019) or worse social functioning (Gonzálvez et al., 2019a,b) have been reported. Taking into consideration the negative consequences related to this problem, it is essential to find variables, such as affect, that can serve as a protective factor of this behavior. In this study, findings suggest the relevance of developing more adaptative affective profiles, such as the self-fulfilling profile, which would contribute to diminishing school attendance problems. Specifically, the data provided by this study suggest that self-destructive and high affective profiles (whose common denominator is high levels of negative affect) are the most maladaptive in this respect. Considering the results, it is important to diminish these levels of negative affect in students by means of techniques or strategies that have shown to be beneficial for positive affect and detrimental for negative affect. These strategies would include cognitive restructuring, mindfulness, and the promotion of self-esteem and motivation, among others (Shikatani et al., 2014; Gómez-Baya et al., 2018; Galla et al., 2020). Likewise, it would be advisable to use programs such as INTEMO (Ruiz-Aranda et al., 2013), which aims to develop emotional skills in adolescents and has shown positive results in reducing certain attitudes toward school dysfunction and other variables such as anxiety, stress, or depression. All of this would facilitate the development of more adaptive affective profiles, such as the self-fulfilling profile, which would contribute to diminishing school attendance problems.

## Data Availability Statement

The raw data supporting the conclusions of this article will be made available by the authors, without undue reservation.

## Ethics Statement

The studies involving human participants were reviewed and approved by UA-2017-09-05. Written informed consent to participate in this study was provided by the participants' legal guardian/next of kin.

## Author Contributions

CG, AD-H, and AF-S: conceptualization. RS and MV: methodology. RS and CR-E: formal analysis. MV and AF-S: investigation. CG and AD-H: writing—original draft preparation. CR-E: writing—review and editing. All the authors contributed equally to the research design, data analysis, and revision and approved the final manuscript.

## Conflict of Interest

The authors declare that the research was conducted in the absence of any commercial or financial relationships that could be construed as a potential conflict of interest.
